# Inference on chains of disease progression based on disease networks

**DOI:** 10.1371/journal.pone.0218871

**Published:** 2019-06-28

**Authors:** Dong-gi Lee, Myungjun Kim, Hyunjung Shin

**Affiliations:** Department of Industrial Engineering, Ajou University, Yeongtong-gu, Suwon, South Korea; Instituto Nacional de Medicina Genomica, MEXICO

## Abstract

**Motivation:**

Disease progression originates from the concept that an individual disease may go through different changes as it evolves, and such changes can cause new diseases. It is important to find a progression between diseases since knowing the prior-posterior relationship beforehand can prevent further complications or evolutions to other diseases. Furthermore, the series of progressions can be represented in the form of a chain, which enables us to readily infer successive influences from one disease to another after many passages through other diseases.

**Methods:**

In this paper, we propose a systematic approach for finding a disease progression chain from a source disease to a target one via exploring a disease network. The network is constructed based on various sets of biomedical data. To find the most influential progression chains, the *k*-shortest path search algorithm is employed. The most representative algorithms such as A*, Dijkstra, and Yen’s are incorporated into the proposed method.

**Results:**

A disease network consisting of 3,302 diseases was constructed based on four sources of biomedical data: disease-protein relations, biological pathways, clinical history, and biomedical literature information. The last three sets of data contain prior-posterior information, and they endow directionality on the edges of the network. The results were interesting and informative: for example, when colitis and respiratory insufficiency were set as a source disease and a target one, respectively, five progression chains were found within several seconds (when *k* = 5). Each chain was provided with a progression score, which indicates the strength of plausibility relative to others. Similarly, the proposed method can be expanded to any pair of source-target diseases in the network. This can be utilized as a preliminary tool for inferring complications or progressions between diseases.

## Introduction

An individual disease may go through different changes as it evolves, and such changes can cause new diseases [1]. The concept of progression between diseases came across as a form of complications or sequelae from a disease. For example, insulin resistance can cause diabetes mellitus type 2 [2], and it can further lead to chronic kidney disease [3, 4]. It is important to find disease progressions since knowing the prior-posterior relationship beforehand can prevent further complications or progressions to other diseases. Progression between diseases has long been studied through cohort verification between two diseases [2, 5–7]. Although the results provided valuable information concerning disease progressions, the time and cost to obtain the results were rather expensive. In recent years, researches have been conducted to find causality between diseases by utilizing diversified biomedical data. In [8], the authors proposed a model for causality using gene/protein, clinical, and metabolic pathway information to construct a disease causality network. In [9], a text mining approach was employed on biomedical literature to construct a causal disease network. Note that causality of disease in these researches stands for potential progression/evolution of various diseases, not the underlying causes of diseases.

Extending from the concept of disease progression, there may be a continuous path or series of diseases that reflect a prior-posterior relation between two diseases. For instance, it is difficult to find a prior-posterior relation between colitis and respiratory insufficiency if we simply look at them directly. However, colitis can progress acute kidney injury, which can lead to polyuria, then to hyponatremia, and finally to respiratory insufficiency. In this paper, we define such a chain of relations among diseases as a disease progression chain. As in the case of colitis and respiratory insufficiency, a disease progression chain can extract prior-posterior relations between two diseases that are seemingly unrelated on the surface.

There were some previous studies concerning diseases in perspectives of chain [1, 10–14]. They defined a causal chain that focused on pathological viewpoints in which diseases are caused by the series of risk factors such as abnormal states, symptoms, or lifestyles. In this research, instead of taking a pathological viewpoint, we attempt to find a disease progression chain in the view of pan-disease, which relates to various diseases with prior-posterior relations. From the clinical viewpoint, disease progression chains can be beneficial for developing continuity of treatment by retrospectively tracing back through the series of diseases. Furthermore, disease progression chains yield a wider angle of disease-related genes (proteins) to consider with the inclusion of diseases composing the chain. The chains may uncover other disease-related genes associated with a specific disease through series of relationships that can be helpful for drug discovery or repositioning. In [15], the authors attempted to detect new target disease of drug considering similar side-effect between diseases, and Chiang and Butte [16] suggested the use of same drug for diseases with similar therapies in terms of disease relationships.

One effective and efficient approach to finding a disease progression chain is to utilize network modeling. Using a network analysis of data has the advantage of scrutinizing relations between data from a more comprehensive and systematic point of view. In constructing a disease network, nodes represent disease, and edges represent genetic, biological, pathological, epidemiological, or other relations between diseases [17–21]. Many researches that utilizing disease network analysis have been carried out in biomedical field to such as establishing genotypes and phenotypes of diseases [22, 23], identifying disease-related genes [24] and drug target genes [25], and repurposing drugs [26].

In addition, if we extend associative relations to prior-posterior relations between diseases, the network is a directed network that can be regarded as a disease progression network. From the constructed network, a simple approach to find a disease progression chain is to manually track the diseases by considering all the possible cases. This approach, however, is very unrealistic owing to a very high number of cases to consider. One possible solution to this issue is to use a shortest path search algorithm, a well-established approach in network (graph) theory. The shortest path search algorithm is a method of searching the path with the lowest cost (or the highest profit) from the source node to the target node. Given a directed network (graph) with nodes and weighted edges, it finds the best single path from various possible paths between two nodes, the shortest path if the edge-weights are defined as distances or the strongest path if edge-weights represent similarities. Applied to disease progression networks, it finds the most probable paths that the largest number of genes shared by diseases, the most frequently co-occurring diseases from clinical information, and the most confirmative relationship of diseases from related researches. Each of networks in the order will be presented in the sub-sections of construction of disease progression networks. The most representative algorithms [27] are the A* algorithm [28], Dijkstra algorithm [29], and Floyd-Warshall algorithm [30, 31]. These algorithms, however, concentrate on finding the shortest path, whereas there could be other “short” paths that contain meaningful information. For this research, we employ the idea of the *k*-shortest path search algorithm, which was first suggested by Yen [32]. The original Yen’s algorithm first finds the shortest path and searches for *k—*1 consecutive shortest paths by eliminating the edges of the shortest path. Many researches have been carried out to improve the complexity of Yen’s algorithm [33–35]. The *k*-shortest path search algorithm has been used in various researches regardless of the field. In [36], the authors applied the *k*-shortest path search algorithm for public transport travel optimization. From the *k*-shortest paths, they selected the optimal path based on the preferences of users. In [37], safe paths in vehicle navigation were recommended based on a risk model that considered crime incidents in an urban road network. To calculate numerous safe paths, the *k*-shortest path search algorithm was applied. Other applications of the *k*-shortest path search algorithm include adjusting traffic flows from overloaded links to underutilized links in a telecommunication network [38] and detecting objects in individual frames of a video [39, 40]. Likewise, there have been numerous researches employing the shortest path search algorithm in the bioinformatics area. In [41–43], the shortest paths in a protein-protein interaction (PPI) network were calculated to find genes that were related to diseases. In [44], the authors defined the mechanism of Parkinson’s disease by identifying genes, miRNA, and potential drug targets using the shortest path search algorithm to determine a microarray gene expression dataset. In [45], regulatory pathways were inferred from a gene network with the shortest path search algorithm, and the same objects exhibiting slight variations in bioimages were analyzed with shortest path search algorithm [46].

In this paper, we propose a systematic approach to find the disease progression chain between two diseases by using a disease progression network constructed from various biomedical data. To find the chains between two diseases, we devise a *k*-shortest path search algorithm that combines the A* algorithm, Dijkstra algorithm, and Yen’s algorithm. Instead of identifying the single shortest path, it is desirable to find the *k*-shortest paths; there may be many different paths in the disease progression network between two diseases. Such consecutive paths may also contain meaningful information on disease progression chain. The rest of the paper is organized as follows. In the proposed method section, we explain the step-by-step process of constructing the disease progression network and finding disease progression chains. In the experiments section, we present experiments and results of applying the proposed method to various biomedical data. In the conclusions section, we conclude the paper with insights and future works of study.

## Proposed method

**Fig 1:**
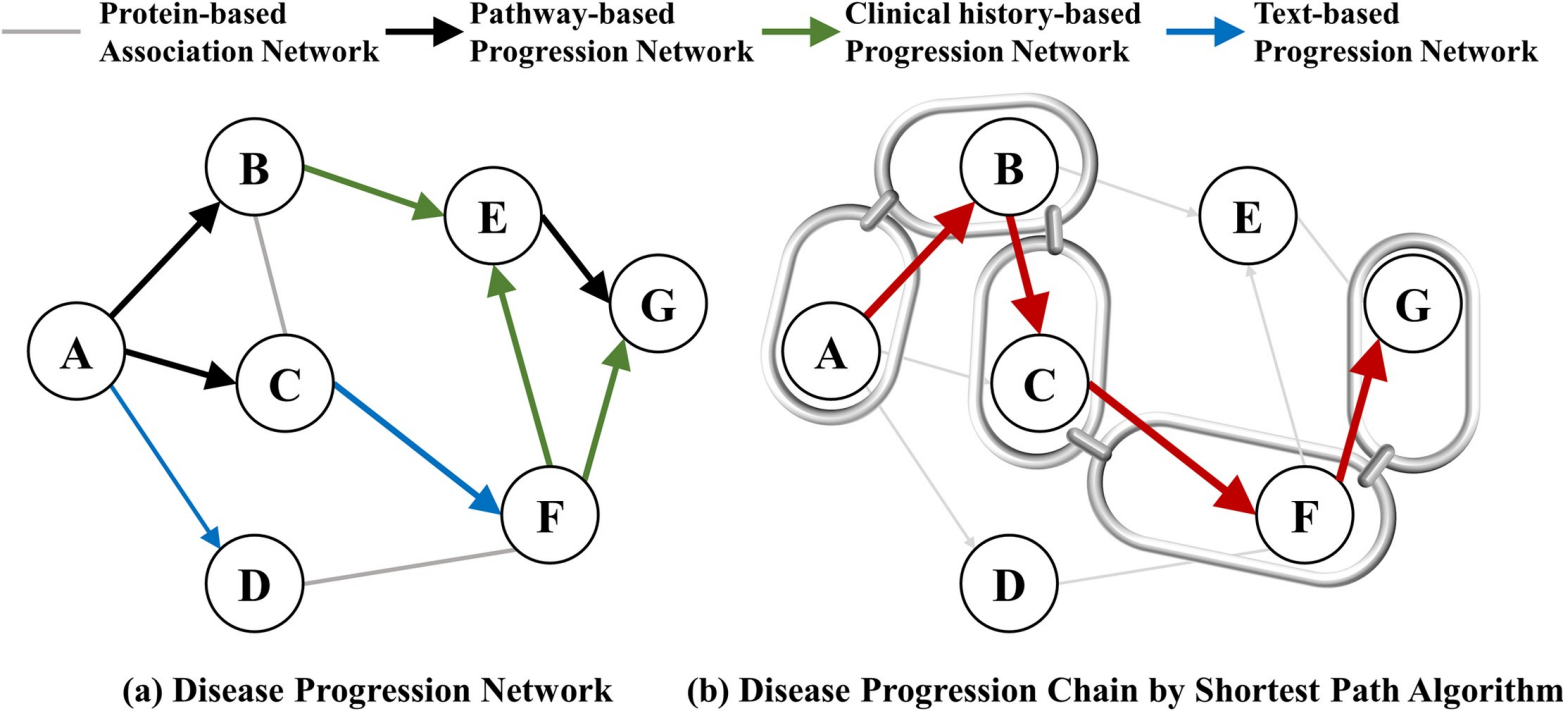
Schematic description of the proposed method: (a) Disease progression network. (b) Disease progression chain by shortest path algorithm.

The proposed method consists of two steps. First, disease progression networks are constructed based on various biomedical data that are related to diseases. The information includes disease-protein relations, biological pathways, clinical history, and biomedical literature. Four networks, each constructed from different information, are integrated into a single network. From the integrated network, we employ the *k*-shortest path search algorithm to find disease progression chains that have the most influence on prior-posterior relations between two diseases. Fig 1 shows a schematic description of the proposed method.

### Construction of disease progression networks

#### Association disease network

**Fig 2:**
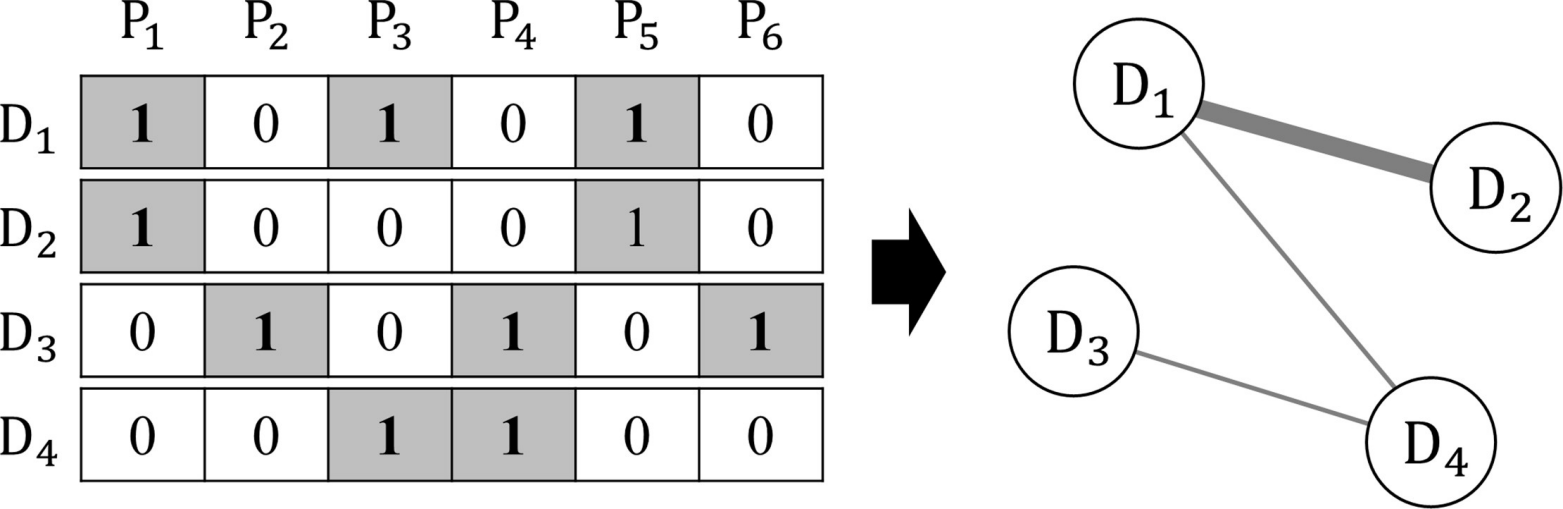
Example of calculating similarity between diseases in ADN.

An association disease network (ADN) is the most fundamental disease network that is constructed based on disease-protein relations. The relation is represented by a bit vector where each bit indicates the existence of relations of a protein to the disease. To quantify the degree of relation between diseases, the cosine similarity between two vectors is used. For diseases *d*_*i*_ and *d*_*j*_ in an ADN, the weight $w_{ij}^{A}$ is calculated by

$$w_{ij}^{A}=\frac{d_{i}{\cdot d}_{j}}{\|d_{i}\|\cdot \|d_{j}\|}$$ (1)

where $0\leq w_{ij}^{A}\leq 1$ and has a higher value for higher numbers of shared proteins between *d*_*i*_ and *d*_*j*_. Fig 2 shows an example of the computing similarity for an ADN.

### Pathway-based disease progression network

To construct a pathway-based disease progression network (pDPN) based on biological pathway information, we employ the method in [8] where prior-posterior relation between two diseases is derived by analyzing pathways associated with the diseases.

**Fig 3:**
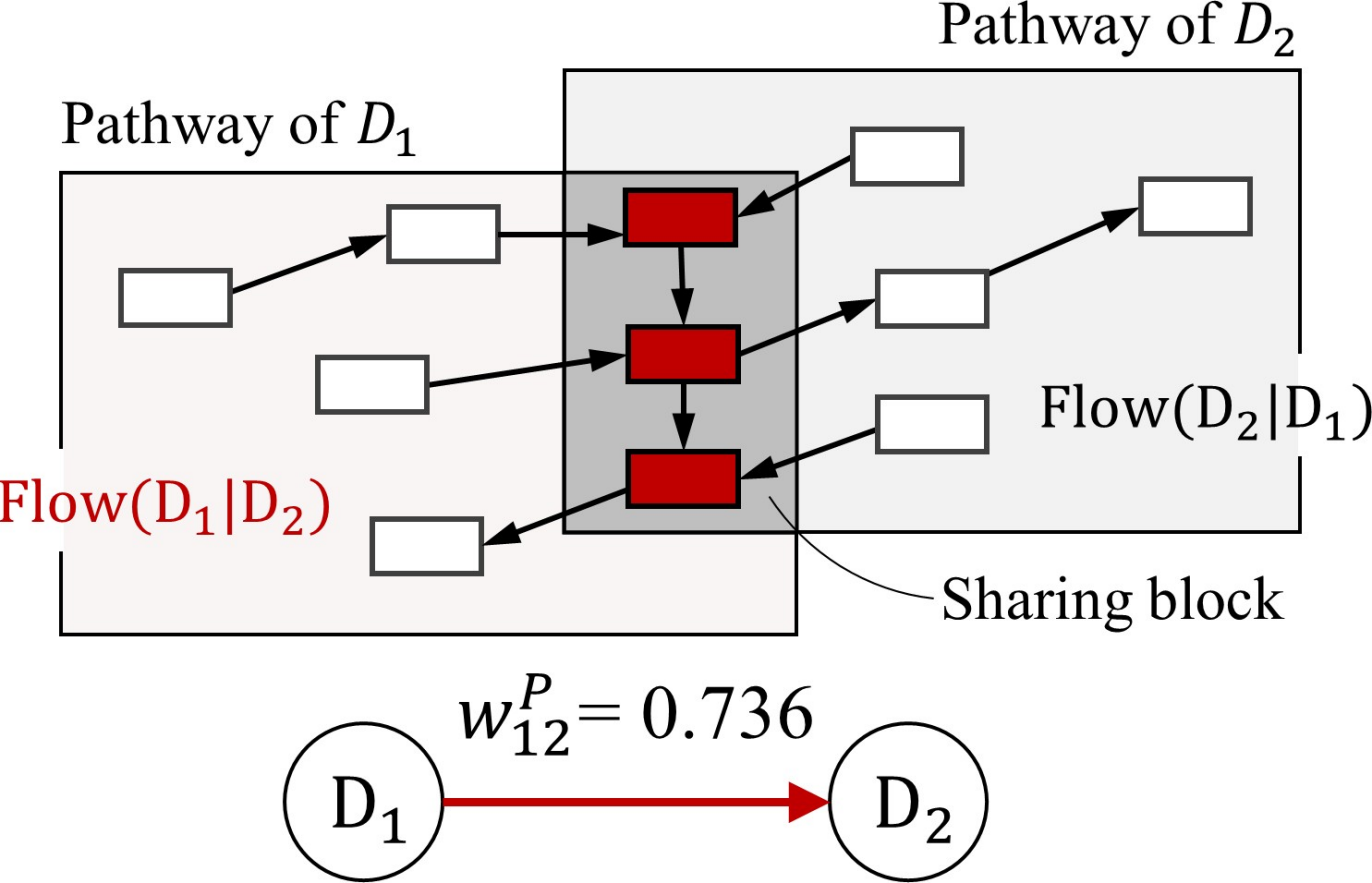
Example of calculating weight between diseases in pDPN.

First, common genes that are included in the pathways of two diseases are extracted and defined as a sharing block. Additionally, a flow function that quantifies the degree of which one disease affects the other disease is defined. The flow function considers the direction of molecular genes that are not included in the sharing block. That is, Flow(*d*_*i*_|*d*_*j*_) is equal to the number of molecular reactions directed toward disease *d*_*j*_ from disease *d*_*i*_, and Flow(*d*_*j*_|*d*_*i*_) is equal to the opposite. To determine the prior-posterior relation, we compare the two flow values and set the weight matrix with

$$w_{ij}^{P}=\varphi \left(Flow(d_{i}|d_{j})-Flow(d_{j}|d_{i})\right)∙\max\left\{Flow(d_{i}|d_{j}),Flow(d_{j}|d_{i})\right\}$$ (2)

where $\varphi (u)=\left\{ \begin{array}{l} 1,\;if\;u>0\\ 0,\;otherwise \end{array}\right.$. The resulting weight matrix is an asymmetric matrix that defines prior-posterior relation in the direction of high to low flow values. In the same manner, we calculate the weights for all pairs of diseases with known pathway information and construct a pDPN. Fig 3 shows an example of constructing a pDPN.

### Clinical history-based disease progression network

A clinical history-based disease progression network (cDPN) is constructed based on the concept of relative risk. Relative risk is an index that describes the association between risk factors and incidents. The relative risk (RR) is given as

$$RR(A,B)=\frac{p(B|A)}{p(B|∼A)}.$$ (3)

where *RR*(*A*,*B*)>1 implies that A influences B with a prior-posterior relation. In the same way, *RR*(*B*,*A*) can also be calculated. For a cDPN, to calculate the associated probabilities for two diseases *d*_*i*_ and *d*_*j*_, we use the number of patients who carry only one or both of *d*_*i*_ and *d*_*j*_. Thus, the progression network is constructed with RR obtained from clinical information.

**Fig 4:**
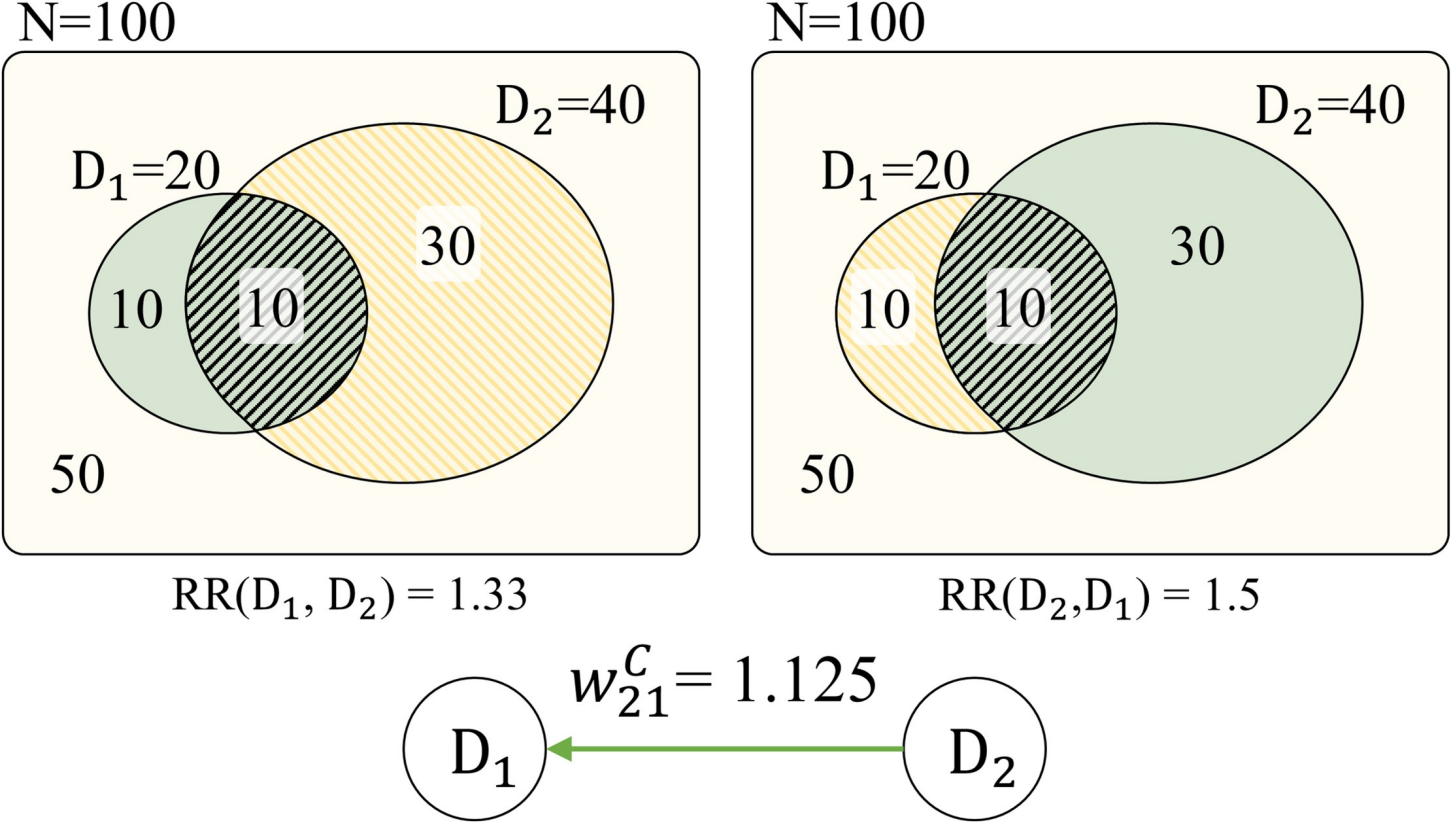
Example of calculating weight between diseases in cDPN.

To determine the prior-posterior relation between *d*_*i*_ and *d*_*j*_, the ratio of relative risk (RRR) that compares the two RR values is calculated. With RR and RRR, the weight $w_{ij}^{C}$ between *d*_*i*_ and *d*_*j*_ in the cDPN is calculated with the following:

$$w_{ij}^{C}=\varphi \left(RR(d_{i},d_{j})-RR(d_{j},d_{i})\right)∙RRR(d_{i},d_{j})$$ (4)

where $RRR(d_{i},d_{j})=\frac{RR(d_{i},d_{j})}{RR(d_{j},d_{i})}$. This approach of defining prior-posterior relation based on clinical history was introduced in [8]. Fig 4 illustrates an example of constructing a cDPN.

### Text-based disease progression network

For a text-based disease progression network (tDPN), we extract the prior-posterior relation between two diseases from text data, and quantify the information [9]. To construct a disease progression network, we consider the following two aspects. First, terms that represent a prior-posterior relation between diseases are defined, and the degree of strength is assigned based on interpretations. Second, clauses that appear in multiple documents should have more influence on prior-posterior relation than clauses that appear multiple times in a single document.

The degree of strength in which terms that represent a prior-posterior relation is defined by *α*_*t*_. The value *α*_*t*_ has higher weight if the term *t* has a stronger implication on prior-posterior relation, and the value has a lower weight if the term simply implies association. For a frequency-based approach, the strength is calculated by considering the number of documents that expresses prior-posterior relation using the term *t* $\left({df}_{t}^{ij}\right)$ and the number of clauses appearing across the documents (${cf}_{t}^{ij}$). The relation strength between diseases *d*_*i*_ and *d*_*j*_ in the text data with a document-clause frequency (DCF) is given by

$${DCF}_{t}^{ij}={df}_{t}^{ij}∙\log\left({cf}_{t}^{ij}+1\right).$$ (5)

**Fig 5:**
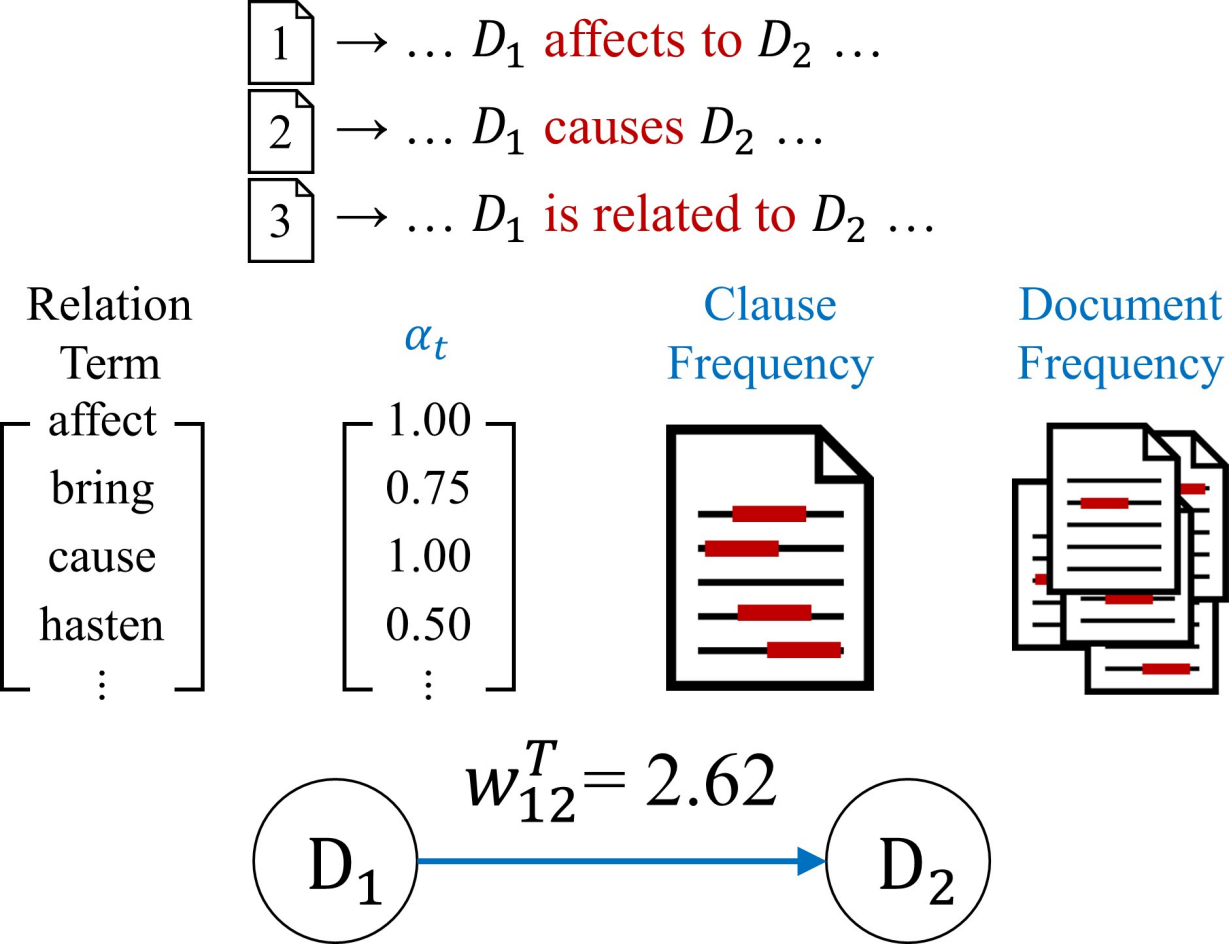
Example of calculating weight between diseases in tDPN.

To define the prior-posterior relation between *d*_*i*_ and *d*_*j*_, the strength of term *α*_*t*_ and ${DCF}_{t}^{ij}$ are combined, and the cases *d*_*i*_→*d*_*j*_ and *d*_*j*_→*d*_*i*_ are compared as in (6) to determine the weight value and its direction.

$$w_{ij}^{T}=\psi (s_{ij}-s_{ji})$$ (6)

where $\psi (u)=\left\{ \begin{array}{l} u,\;if\;u>0\\ 0,\;otherwise \end{array}\right.$ and $s_{ij}=\sum_{t\in T}\left(\alpha_{t}^{ij}∙DCF_{t}^{ij}\right)$, and *s*_*ij*_ denotes the strength of prior-posterior relation when disease *d*_*i*_ affects disease *d*_*j*_. Fig 5 shows an example of constructing a tDPN from text data.

### Chain of disease progression

#### Search algorithm for progression path

To find a disease progression chain, the *k*-shortest path search algorithm is utilized. Simple shortest path search algorithms only search for a single path. In the problem of finding a disease progression chain, one disease may lead to the other through many different paths in the disease progression network. Thus, it is desirable to find the *k*-shortest paths instead of the single shortest path. In this paper, the *k*-shortest paths for two diseases in a DPN is found by using a modified version of Yen’s algorithm [32]. Given a graph, Yen’s algorithm first finds the shortest path between two nodes and searches for consecutive shortest paths by enlarging the values of edges that are part of the shortest path. The method of searching the paths is based on the Dijkstra algorithm [29], a greedy search algorithm for finding the shortest path. In this study, we modify Yen’s algorithm by using a combination of the Dijkstra algorithm and A* algorithm [28] in search of the shortest paths. This combination can reduce the computational time compared to the Dijkstra algorithm, and guarantees optimality [42].

To briefly review, suppose there is a graph *G*(*D*,*E*) where *D* = {*d*_1_,*d*_2_,…,*d*_*n*_} is the set of nodes and *E* denotes the set of weighted edges, possibly with directions. The Dijkstra algorithm starts by setting the initial starting node, say *d*_*S*_, and stores the vertex with the distance at each iteration. If a stored vertex is reached from another path, the distance is updated with the smaller value. Through the iterations, the shortest path from *d*_*S*_ to every element in {*d*_*j*_:*d*_*j*_∈*D*,*d*_*j*_≠*d*_*S*_} is obtained. The principle of the A* algorithm is similar to that of the Dijkstra algorithm except that it uses an evaluation function:

$$f(d_{v})=g(d_{v})+h(d_{v})$$ (7)

where *g*(*d*_*v*_) is the minimum distance possible from *d*_*S*_ to *d*_*v*_, and *h*(*d*_*v*_) is the estimate of the cost of an optimal path from *d*_*v*_ to the target node *d*_*T*_. From the starting node *d*_*S*_, the A* algorithm searches for the smallest *f* at each iteration until *d*_*T*_ is reached. When *h* = 0 for all *d*_*v*_∈*V*, then the A* algorithm is equivalent to the Dijkstra algorithm.

The combination of the Dijkstra algorithm and A* algorithm is given as follows. First, the Dijkstra algorithm is applied to the DPN in the reverse manner. That is, the search starts from the target *d*_*T*_, traces the edges back to their original vertices, and stores each optimal distance *δ*(*d*_*v*_,*d*_*T*_). Then, we set *h*(*d*_*v*_) to be the optimal distance from *d*_*v*_ to *d*_*T*_ obtained from the previous step. Finally, the A* algorithm is applied to search paths from *d*_*S*_ to *d*_*T*_ to find the optimal path. To find the *k*-shortest paths *P*^1^,*P*^1^,…,*P*^*k*^, the following procedure is applied:

1. Apply the A* search algorithm to obtain *P*^1^, the shortest path from *d*_*S*_ to *d*_*T*_. Set *i* = 2.
2. For each edge in *P*^*i*−1^, we cut it and apply the A* search algorithm to obtain the set of potential paths $Z^{i}=\{z_{1}^{i},z_{2}^{i},\ldots ,z_{q}^{i}\}$, where *j* = 1,…*q*, and *q* is the number of edges in *P*^*i*−1^. We discard $z_{j}^{i}$ if $z_{j}^{i}\in {\left\{P^{x}\right\}}_{x=1}^{i-1}$.
3. The shortest path from the set of potential paths becomes *P*^*i*^. Set *i* = *i*+1.
4. Repeat (2) and (3) until *i* = *k*+1 or no potential path can be found.

**Fig 6:**
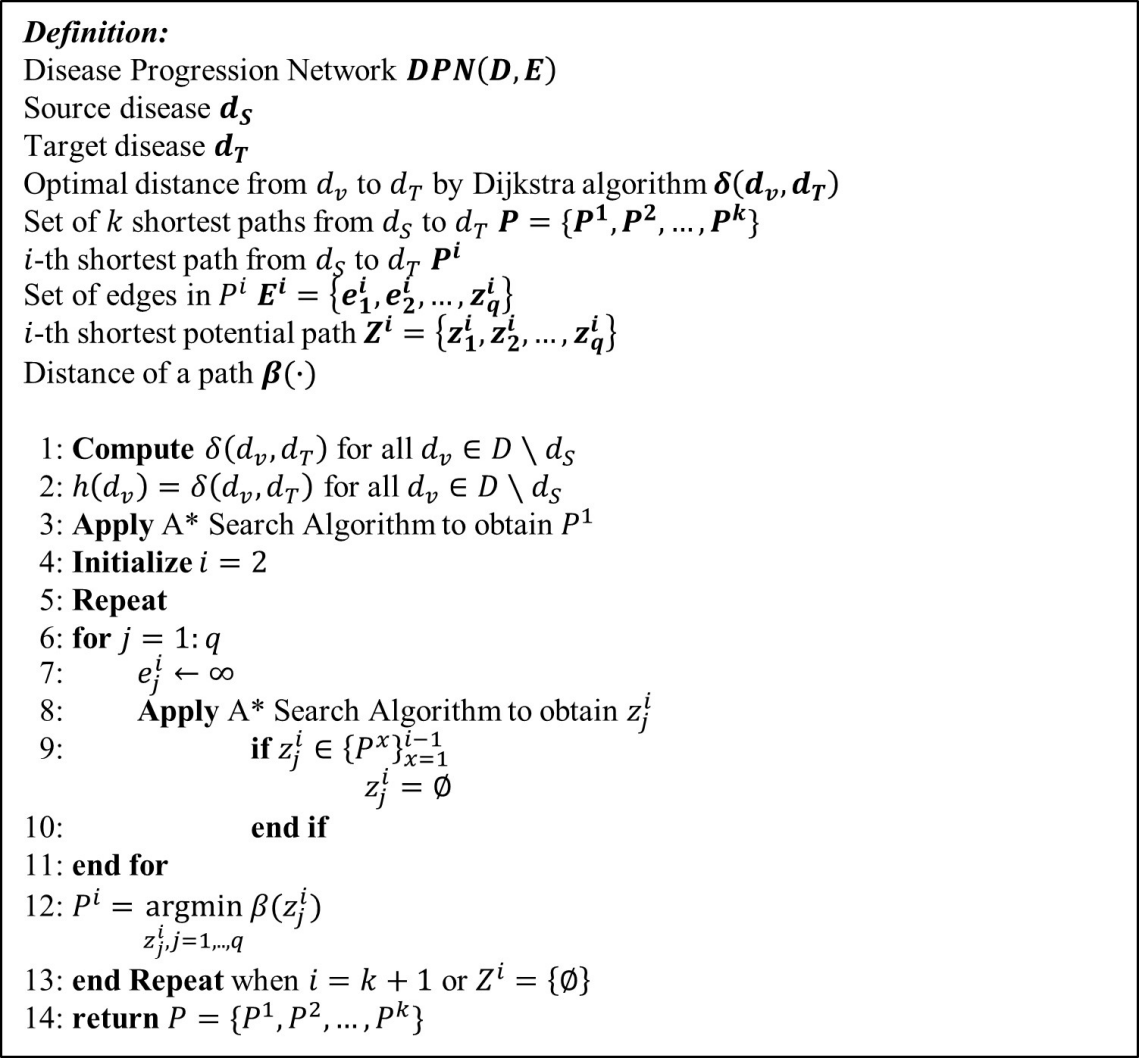
Pseudocode for finding *k*-shortest paths.

The pseudocode for finding the *k*-shortest paths is given in Fig 6.

#### Disease chains and progression scores

The edges in a DPN have weight values between 0 and 1. If the weight between two diseases is high, then it implies a strong casual relation between the two. The shortest path search algorithm has priority in searching edges with low weight values. To reflect this property, the weight values are tranformed into distance values before applying the shortest path search algorithm. The distance between diseases *d*_*i*_ and *d*_*j*_ is defined as

$$dist(d_{i},d_{j})=1-w_{ij}^{INT}$$ (8)

where $w_{ij}^{INT}$ is the edge weight between diseases *d*_*i*_ and *d*_*j*_ in the integrated network. In addition, in contrast to the other three DPNs, edges in an ADN represent association instead of prior-posterior relations between diseases. In the process of finding chains, the bidirected edges in the integrated network are penalized.

By applying the disease progression chain finding algorithm to the integrated network, it is possible to find chains between two diseases of interest. Then, the influence of the chains is measured by the progression score (PS), which quantifies the relative importance of each chain. For the list of diseases in the progression chain between disease A and B, DPC_d_ = {*d*_*A*_,*d*_1_,…,*d*_*n*_,*d*_*B*_}, the importance of each connection in the chain can be obtained by the weighted values in the original integrated network. For the set of weight values in a disease progression chain, DPC_w_ = {*w*_*A*1_,…,*w*_*nB*_}, the PS between disease A and B is calculated by

$$PS(d_{A},d_{B})=\delta \cdot \exp\left(-l+\sum_{w\in DPC_{w}}w\right)$$ (9)

where *l* is the length of the disease progression chain, and *δ* is a scale parameter. Since the weight values lie between 0 and 1, the PS decreases with a greater number of connections between two diseases of interest. In other words, PS depends on the length of the progression chain, where a shorter length implies a larger influence with a more direct relation.

## Experiments

### Data

To implement the proposed method, data on diseases, disease-protein relations, biological pathways, clinical history, and biomedical literature were used. The list of diseases was collected from Medical Subject Headings (MeSH) [47], where MeSH 2018 contains the names of 4,798 diseases in the diseases category. For disease-protein relations, data was collected from PharmDB [48], which provides information on diseases, drugs, and proteins. The data in PharmDB are extracted from various databases including the Comparative Toxicogenomics Database (CTD) [49], Genetic Association Database (GAD) [50], Online Mendelian Inheritance in Man (OMIM) [51], and Pharmacogenomics Knowledge Base (PharmGKB) [52]. To construct pDPN, biological pathway information was used from KEGG [53]. By matching diseases in KEGG and MeSH, 153 diseases were extracted with 129 having pathway information. For cDPN, we used the HuDiNe database [54], which contains 13 million clinical history records of prevalence and comorbidity information for diseases. Last, abstracts of biomedical literature listed in PubMed [55] were utilized for tDPN construction. Table 1 summarizes the data used for the experiments.

**Table 1:** Data description for experiments.

	Data Sources	Number of Data
**Diseases**	**MeSH:** Medical Subject Headings (http://www.nlm.nih.gov/mesh)	4,798 diseases
**Disease-protein relation**	**PharmDB:** Integrated database for diseases, proteins, and drugs including CTD, GAD, OMIM, PharmGKB (http://www.pharmdb.org)	153,118 relations between 2,727 diseases and 23,022 proteins
**Biological pathway**	**KEGG:** Kyoto encyclopedia of genes and genomes (http://www.genome.jp/kegg/pathway.html)	129 pathways related to 153 diseases
**Clinical history**	**HuDiNe** (http://hudine.neu.edu)	1,692 prevalence and 648,886 comorbidities of 13,039,018 patients
**Biomedical literature**	**PubMed:** US National Library of Medicine, National Institutes of Health (http://www.ncbi.nlm.nih.gov/pubmed)	6,617,833 abstracts

### Results for construction of disease progression networks

Four different disease progression networks (ADN, pDPN, cDPN, and tDPN) were constructed by employing the proposed method on the collected data. In the network construction process, disease terms used in KEGG and HuDiNe are different from those in MeSH, where KEGG has its own terms and HuDiNe has ICD9. Therefore, each disease term was mapped to MeSH based on disease ontology in order to standardize and merge into a single term.

Four different DPNs are integrated into a single network in which the algorithm for finding the disease progression chain is applied. The integrated network has 3,302 diseases with 613,270 prior-posterior relations. Table 2 lists the results of network construction with the properties for four different DPNs and the integrated network.

**Table 2:** Result of construction of progression networks.

Properties	ADN	pDPN	cDPN	tDPN	INT^a^
**Number of diseases**	2,727	146	1,692	149	3,302
**Number of relations**	147,290	5,247	468,285	1,011	613,270
**Network density**	1.98%	24.79%	16.37%	4.58%	5.63%
**Clustering coefficient**	0.304	0.397	0.327	0.235	0.331
**Connected components**	2	1	1	2	1
**Network diameter**	6	5	12	7	8
**Network radius**	1	3	1	1	1
**Avg. number of neighbors**	54.592	71.877	553.528	13.570	328.923

^a^INT represents the integrated network of ADN, pDPN, cDPN, and tDPN.

**Fig 7:**
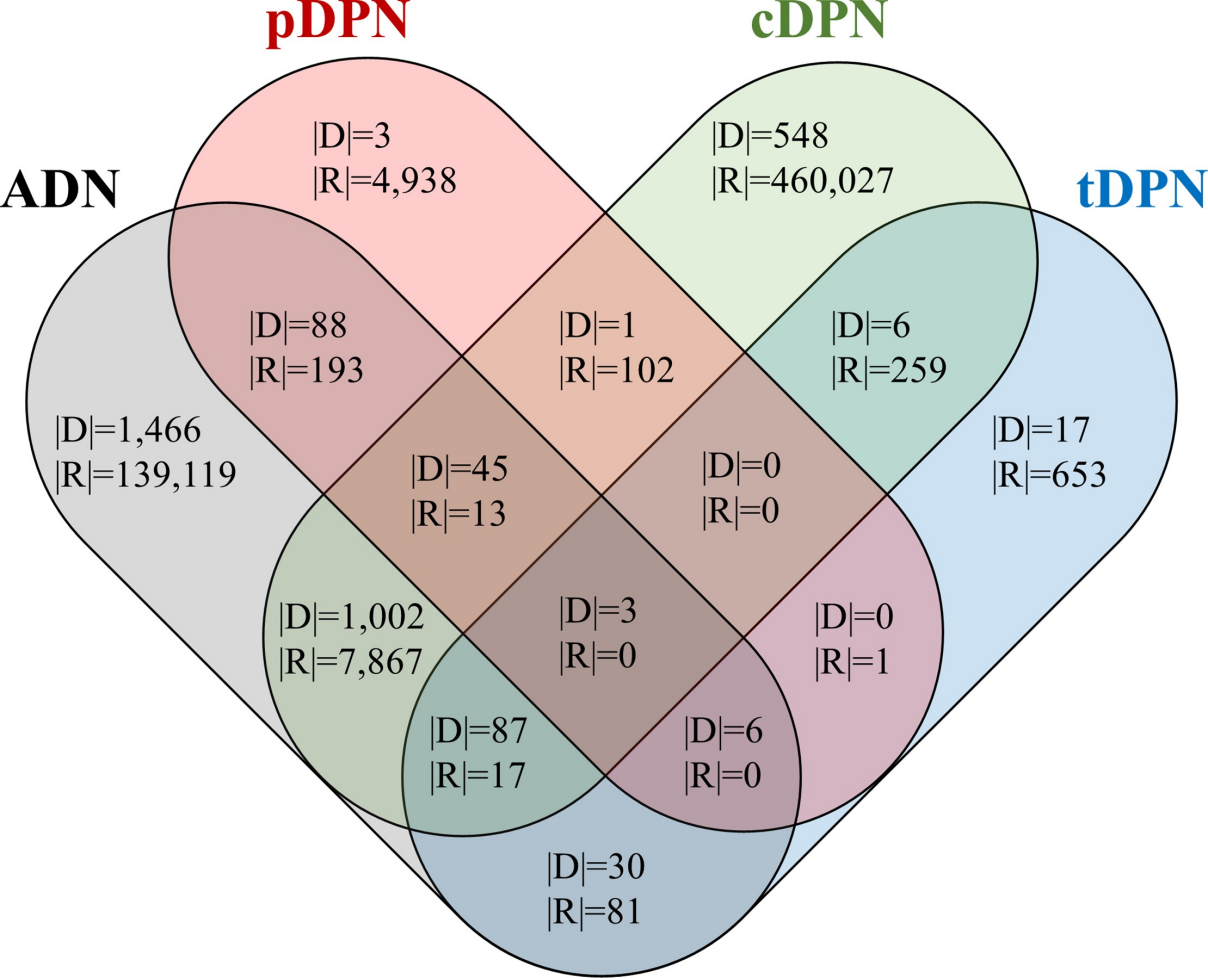
Four-set Venn diagram of overlap of diseases and prior-posterior relations.

Fig 7 is a Venn diagram for ADN, pDPN, cDPN, and tDPN that illustrates the overlap of diseases and prior-posterior relations among different sources. In the figure, |D| is the number of diseases, and |R| is the number of prior-posterior relations.

To outline some characteristics, ADN represents the association between diseases and has the form of an undirected network. In this study, this form is considered a bidirected network that has directions in both ways between two diseases. In addition, from the perspective of a disease progression network, ADN has less significance of disease progression compared to other networks that represent prior-posterior relations instead of association. The abundance of disease-protein relations, however, leads to a relatively dense network. Thus, to construct an ADN with relevant information, the *k*-Nearest Neighbors (*k*-NN) method is applied. In the experiment, when *k* = 40, the density of ADN was reduced from 17.95% (1,334,312 relations) to 1.98% (147,290 relations). In addition, as we can see the Table 2, ADN and cDPN have more diseases and relations compared with pDPN and tDPN. This is due to the difference of inherent characteristics in data sources for constructing each of the networks. For ADN, disease-protein relations have already been established by numerous researches, therefore resulting in high number of diseases and relations. Likewise, the size of data for cDPN is huge with large number of clinical history records. On the other hand, the small size of pDPN is originated from low number of diseases associated with pathways in KEGG. Furthermore, in Fig 7, the overlap of diseases and relations among four networks is scarce. This observation comes from complicated process of linking the biological mechanism, the phenotypes, and the literature knowledge base of diseases.

For the dataset of ADN, pDPN, cDPN, tDPN, and integrated network, refer to S1 Dataset.

### Results for disease progression chain

**Fig 8:**
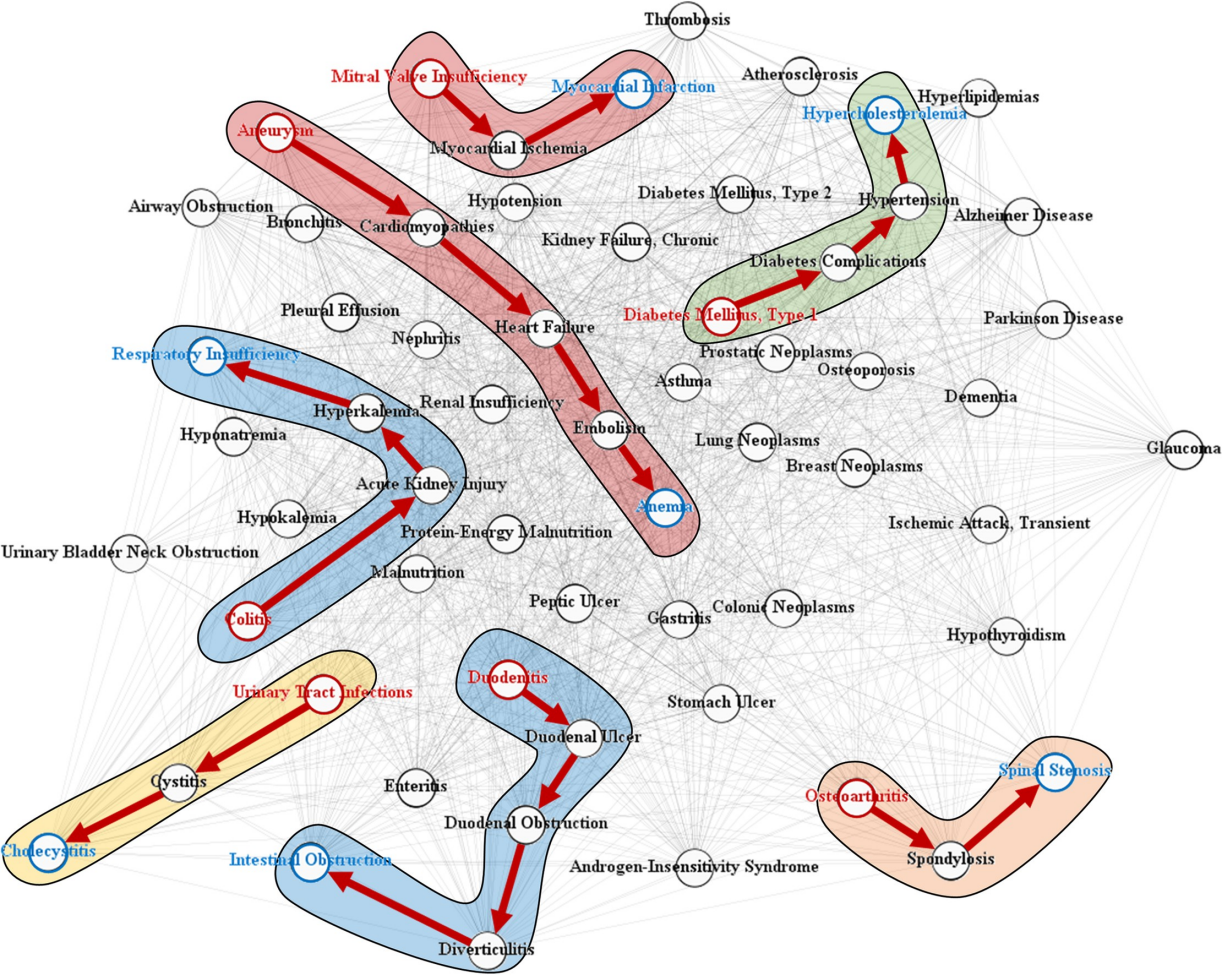
Disease progression chains in integrated disease network.

Fig 8 shows a subset of DPN and some examples of disease progression chains extracted with the search algorithm for progression path. The red and blue nodes represent sources and targets in the disease progression chain, respectively. The backgrounds colored with red, blue, green, yellow, and orange represent cardiovascular, digestive system, metabolic, urogenital, and musculoskeletal diseases, respectively.

**Fig 9:**
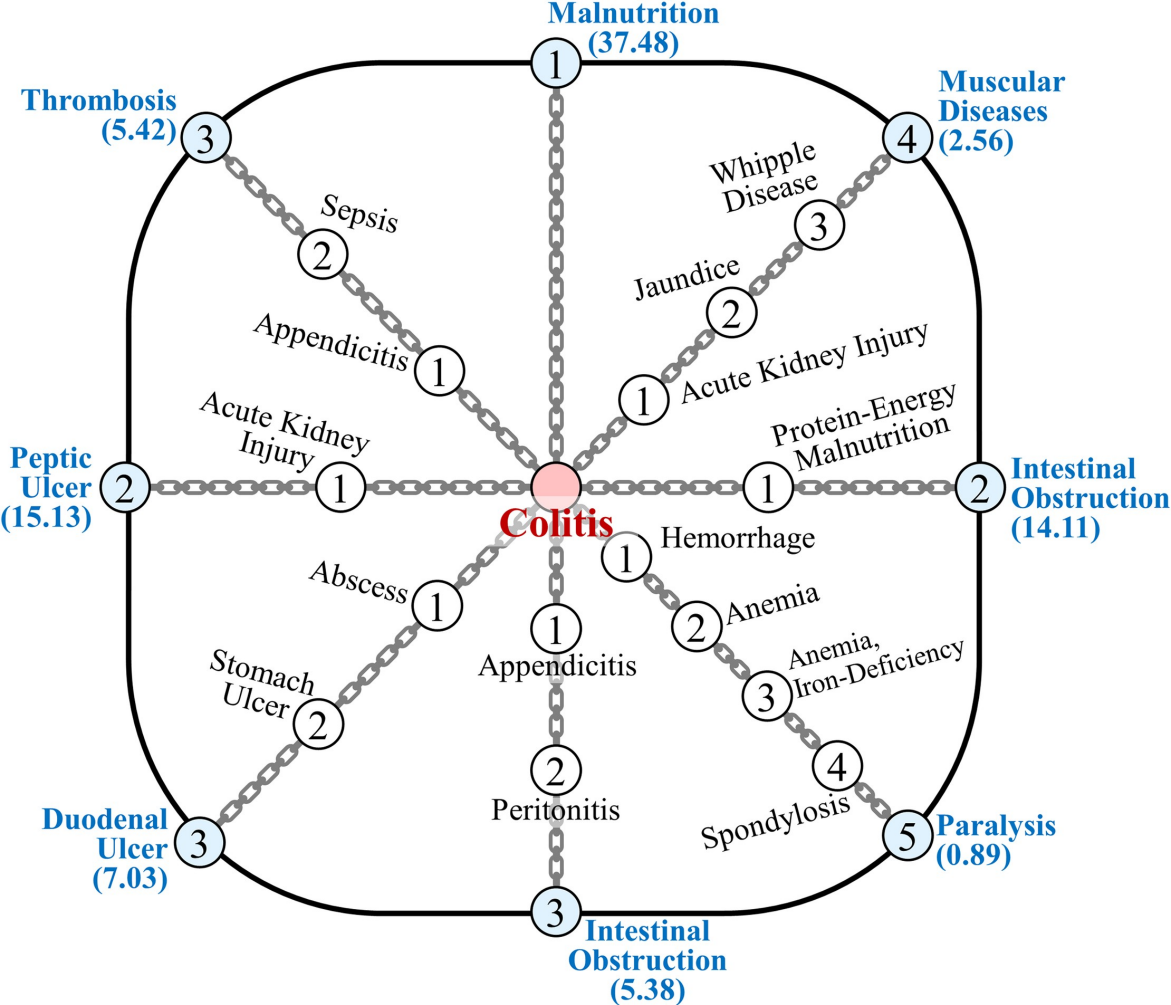
Spectrum of disease progression chains.

Fig 9 shows the disease progression chain spectrum, in which it is possible to determine the process of a particular disease affected by various other diseases. The diseases with blue nodes indicate the destinations of the chains. The number inside the node is the step of the chain, and the value under each disease is the PS.

For the case of colitis, we see various progression chains ranging from the direct connection of malnutrition to paralysis with four bypassing diseases.

### Exploration of disease progression chains in DPN

**Fig 10:**
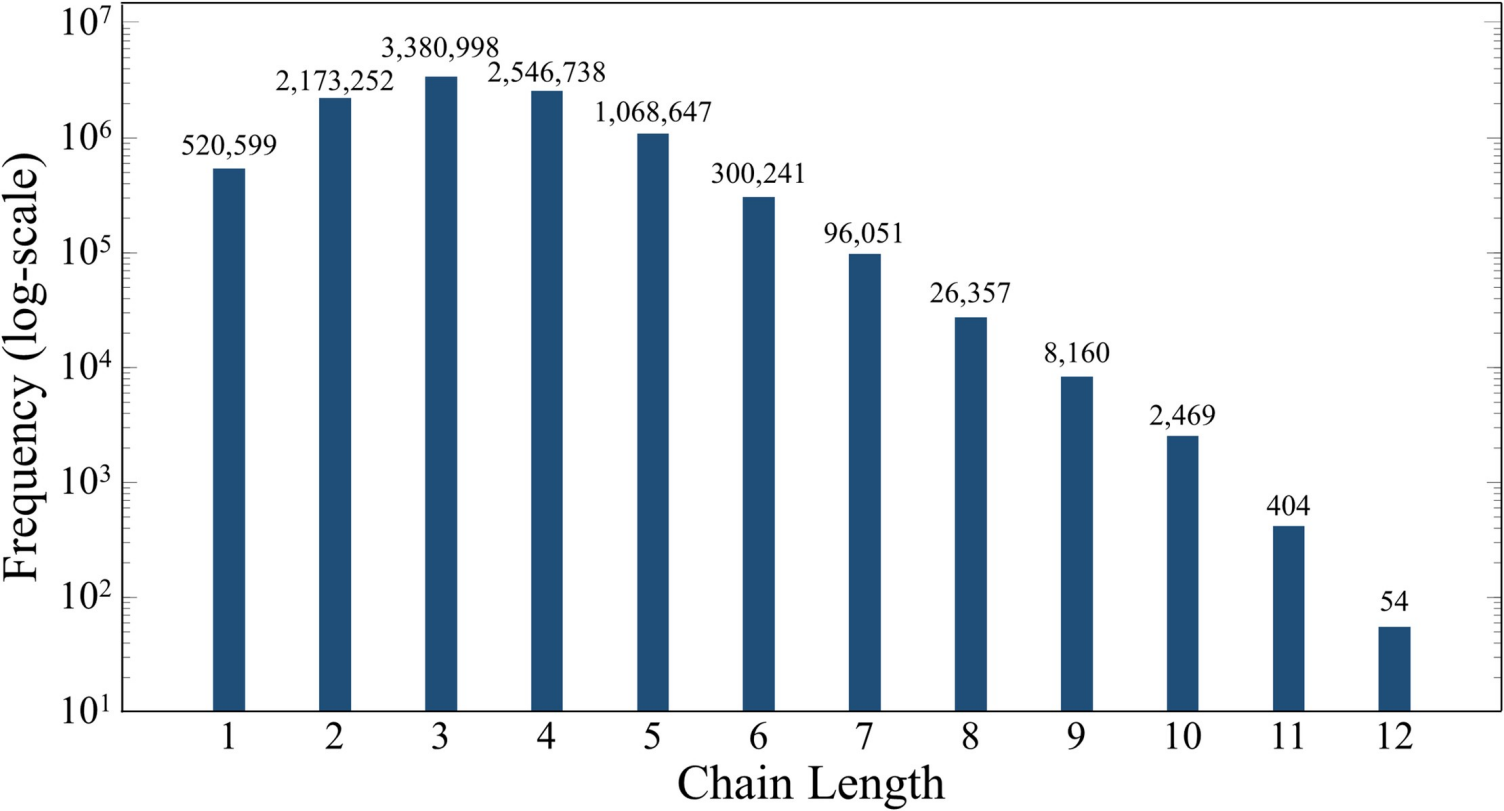
Distribution of disease progression chain lengths among 3,302 diseases.

To examine the overall result of disease progression chains in a disease network, the search algorithm for disease progression chain was applied with *k* = 1 for simplicity. Of 10,899,902 possible pairs, 10,123,970 (92.88%) disease progression chains were extracted. Fig 10 shows the distribution of the number of diseases within the disease progression chain. We see that the chain length varies from one (direct connection) to a maximum of 12 diseases. Furthermore, the disease progression chain has a shorter length for a higher network density, and longer length for a lower network density. This implies that more information in constructing the integrated network leads to closer (direct) relations between diseases in the progression chain.

One example of a disease progression chain with a long length is hypopharyngeal neoplasms and thyroid hormone resistance syndrome. The disease progression chain is given as follows:

[Hypopharyngeal Neoplasms] → [Trigeminal Nerve Injuries] → [Facial Injuries] → [Enophthalmos] → [Trichiasis] → [Trachoma] → [Cataract] → [Macular Degeneration] → [Thyroiditis] → [Thyroid Hormone Resistance Syndrome]

In the disease progression chain, connections from hypopharyngeal neoplasms to trachoma and macular degeneration to thyroiditis are based on clinical history, from trachoma to cataract is based on the biomedical literature, from cataract to macular degeneration is based on biological pathways, and from thyroiditis to thyroid hormone resistance syndrome is based on disease-protein relations.

### Implication of k-chains

The results of *k*>1 are explained with an example of the relations between colitis and respiratory insufficiency. In general, it is difficult to find a direct association between colitis and respiratory insufficiency. By applying the proposed method to corresponding diseases with *k* = 5, we found the following results for the disease progression chain:

1. [Colitis] → [Acute Kidney Injury] → [Polyuria] → [Hyponatremia] → [Respiratory Insufficiency] *(PS = 26*.*31)*
2. [Colitis] → [Diabetes Insipidus] → [Polyuria] → [Hyponatremia] → [Respiratory Insufficiency] *(PS = 25*.*92)*
3. [Colitis] → [Polydipsia] → [Polyuria] → [Hyponatremia] → [Respiratory Insufficiency] *(PS = 24*.*68)*
4. [Colitis] → [Acute Kidney Injury] → [Oliguria] → [Diabetes Insipidus] → [Polyuria] → [Hyponatremia] → [Respiratory Insufficiency] *(PS = 4*.*78)*
5. [Colitis] → [Renal Colic] → [Oliguria] → [Diabetes Insipidus] → [Polyuria] → [Hyponatremia] → [Respiratory Insufficiency] *(PS = 4*.*39)*

From the resulting chains, there is a common path from polyuria to respiratory insufficiency, which passes through hyponatremia. The difference between the chains comes from various paths from colitis to polyuria. For the common path, a close relationship between polyuria and hyponatremia was shown in numerous clinical reports (PMID: 23837469, 10468901), where both diseases were affected by vasopressin. From hyponatremia to respiratory insufficiency, the former reduces cerebral blood flow and arterial oxygen content, which leads to hypoxia and respiratory insufficiency (PMID: 14605269). In addition, it was also reported that hyponatremia can cause a sudden respiratory insufficiency (PMID: 3713746).

In the case of the first chain, it was reported several times (PMID: 23445618, 25056300) that acute kidney injury can be caused by colitis. From acute kidney injury to polyuria, there have been research studies (PMID: 877851, 20525977) that examined the mechanism of a former disease leading to the latter. In a similar approach, it is possible to verify the prior-posterior relations for all five chains. The overall result is shown in Table 3.

**Table 3:** Verification of disease progression chains.

Prior-posterior relations	Verification
Polyuria → Hyponatremia	PMID: 23837469, 10468901
Hyponatremia → Respiratory Insufficiency	PMID: 14605269, 3713746
Colitis → Acute Kidney Injury	PMID: 23445618, 25056300
Acute Kidney Injury → Polyuria	PMID: 877851, 20525977
Colitis → Diabetes Insipidus	PMID: 1582604
Diabetes Insipidus → Polyuria	PMID: 23240316, 28645353
Colitis → Polydipsia	PMID: 17404867
Polydipsia → Polyuria	PMID: 24490488
Acute Kidney Injury → Oliguria	PMID: 21716258
Oliguria → Diabetes Insipidus	PMID: 2929392

With the disease progression chains, we can track numerous cases that describe the process of a disease developing to another from colitis to respiratory insufficiency. For more diverse results and details of the *k*-chains, refer to Table A in the S1 Appendix.

### Validation of disease progression chains

To evaluate the confidence of the proposed method, resulting disease progression chains were validated by comparing them with clinical histories. The prior-posterior relations of diseases with the ratio of relative risk (RRR) from clinical history data contain directions for a significant number of diseases and are based on the information of patients. Although it is the best compare with trajectories referred to patients, it takes tremendous time and effort. In terms of practicality, information on clinical history can serve as the standard for validation. The validation process was carried out as follows: (a) In the integration step, use ADN, pDPN, and tDPN, excluding clinical history information. (b) Apply a search algorithm for progression path to each pair of diseases. (c) Calculate RRR for the selected prior-posterior relations of diseases. As explained in clinical history-based disease progression network section, if *RRR*>1, then it is plausible to evaluate the corresponding prior-posterior relation as a correct relation.

**Fig 11:**
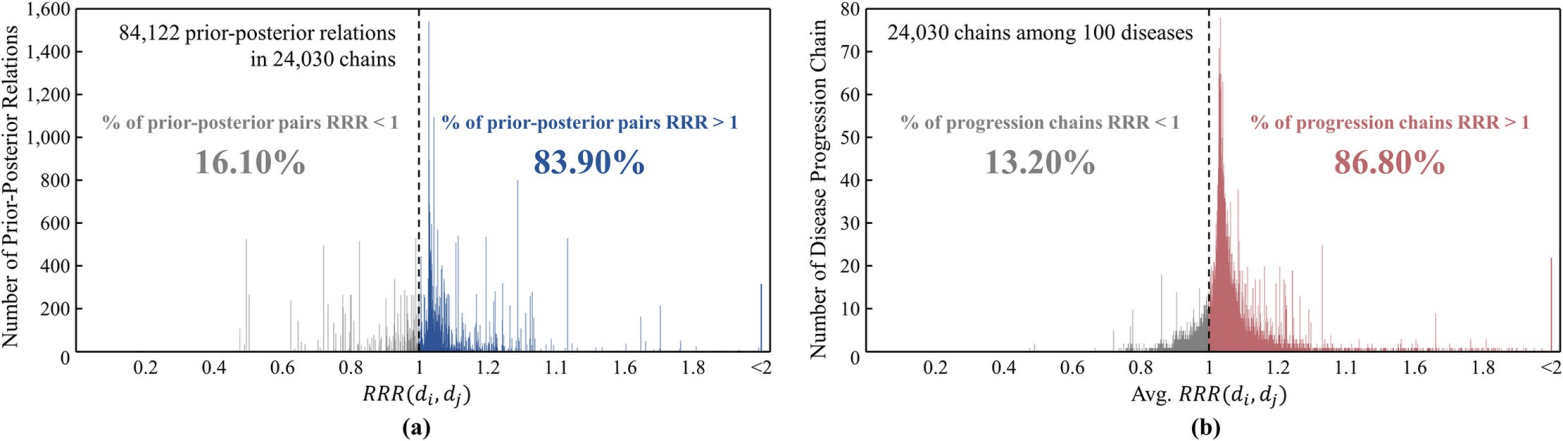
Validation with distribution of ratio of relative risk for prior-posterior relations in disease progression chains: (a) the distribution of RRR of the prior-posterior relations. (b) the distribution of the average RRR for the disease progression chains.

Of 3,302 diseases, 100 were randomly selected, and three chains were found for each disease pair possible. As a result, 24,030 chains were found with 84,122 prior-posterior relations within the chains. The confidence of the proposed method was evaluated based on the ratio of prior-posterior relations with *RRR*>1. Fig 11(A) shows the distribution of RRR of the prior-posterior relations found. The number of prior-posterior relations with *RRR*>1 is 70,575, which corresponds to 83.90% of the total. In addition, the confidence of a disease progression chain can be evaluated based on the average RRR for all prior-posterior relations within the chain. Fig 11(B) shows the distribution of the average RRR for the disease progression chains. The number of chains with average *RRR*>1 is 20,662, which corresponds to 86.80% of the total. The validation results show that the proposed method guarantees high confidence in the results.

## Conclusions

In this paper, we proposed a method of finding a series of disease progressions, the disease progression chain, from an integrated disease progression network constructed with various biomedical data. The disease progression network was constructed by integrating four different sources of disease-protein relation, biological pathway, clinical history, and biomedical literature. To find disease progression chains, a *k*-shortest path search algorithm that combines the A* algorithm, Dijkstra algorithm, and Yen’s algorithm was proposed. Through the proposed method, various disease progression chains between two diseases were found and were verified qualitatively from biomedical literature and quantitatively with comparisons between clinical histories and other sources of information.

The novelty of this research is that the concept of the disease progression chain, proposed in this paper, can be beneficial for tracking the prognosis of various diseases that can follow from an occurrence of a disease. In addition, the prior-posterior relations between two diseases from different categories can also be found despite their seemingly low association. On the other hand, there are some limitations in the present study. For the disease progression networks, each of sources has its domain trait which may be deserved to be preserved. However, the problem is the respective networks are sparse and disconnected. This means we can hardly find a relevant path from a single network, thus the network integration was employed. In addition, it would be better to give the networks different weights according to the relative importance. However, we currently have only a little knowledge on which network is better than the other. Therefore, we treated the networks (except the association network) with same weights in the network integration process. But the performance of the proposed method will be improved if we reweight the networks according to significance of each of source domains. In principle, it will be worth finding the path on disease progression from an individual network not from integrated one when abundant knowledge become more available than now. Moreover, the validation of disease progression chains in the current study was compared with clinical history data, but it would be more thorough if the results are compared to trajectories of diseases referred to patients.

In these respects, this research can further be developed by enriching the disease progression network with more abundant information, integrating networks with different weights according to significance, improving the proposed *k*-shortest path search algorithm, and refining verification methods through comparisons with cohort studies. These will be important aspects of our future work. With improved verification methods, it can benefit the role of therapy and its temporal assessment in the progression of diseases. We hope that the proposed method is perceived as a preliminary information that may help practitioners have some hints that may be far better than beginning from nothing at all.

## Supporting information

S1 Dataset Four individual disease progression networks and the integrated network. (ZIP)

S1 Appendix Supplementary information of disease progression chains. (PDF)

## References

[1] Kozaki K, Mizoguchi R, Imai T, Ohe K, editors. Identity Tracking of a Disease as a Causal Chain. Proceedings of the 3rd International Conference on Biomedical Ontology (ICBO2012); 2012.

[2] Lillioja S, Mott DM, Spraul M, Ferraro R, Foley JE, Ravussin E, et al Insulin resistance and insulin secretory dysfunction as precursors of non-insulin-dependent diabetes mellitus: prospective studies of Pima Indians. New England Journal of Medicine. 1993;329(27):1988–92. 10.1056/NEJM199312303292703 8247074

[3] Bailey RA, Wang Y, Zhu V, Rupnow MF. Chronic kidney disease in US adults with type 2 diabetes: an updated national estimate of prevalence based on Kidney Disease: Improving Global Outcomes (KDIGO) staging. BMC research notes. 2014;7(1):415. 24990184 10.1186/1756-0500-7-415 PMC4091951

[4] van der Meer V, Wielders HPM, Grootendorst DC, de Kanter JS, Sijpkens YW, Assendelft WJ, et al Chronic kidney disease in patients with diabetes mellitus type 2 or hypertension in general practice. Br J Gen Pract. 2010;60(581):884–90. 10.3399/bjgp10X544041 21144198 PMC2991741

[5] Hemingway H, Marmot M. Psychosocial factors in the aetiology and prognosis of coronary heart disease: systematic review of prospective cohort studies. Bmj. 1999;318(7196):1460–7. 10.1136/bmj.318.7196.1460 10346775 PMC1115843

[6] Kukull WA, Higdon R, Bowen JD, McCormick WC, Teri L, Schellenberg GD, et al Dementia and Alzheimer disease incidence: a prospective cohort study. Archives of neurology. 2002;59(11):1737–46. 12433261 10.1001/archneur.59.11.1737

[7] McDonald GB, Hinds MS, Fisher LD, Schoch HG, Wolford JL, Banaji M, et al Veno-occlusive disease of the liver and multiorgan failure after bone marrow transplantation: a cohort study of 355 patients. Annals of internal medicine. 1993;118(4):255–67. 8420443 10.7326/0003-4819-118-4-199302150-00003

[8] Bang S, Kim J-H, Shin H. Causality modeling for directed disease network. Bioinformatics. 2016;32(17):i437–i44. 10.1093/bioinformatics/btw439 27587660

[9] Lee D-g, Shin H. Disease causality extraction based on lexical semantics and document-clause frequency from biomedical literature. BMC medical informatics and decision making. 2017;17(1):53. 28539124 10.1186/s12911-017-0448-y PMC5444051

[10] Friedman GD, Steinberg B. Primer of epidemiology. 1994.

[11] Kozaki K, Kou H, Yamagata Y, Imai T, Ohe K, Mizoguchi R, editors. Browsing causal chains in a disease ontology. Proceedings of the 2012th International Conference on Posters & Demonstrations Track-Volume 914; 2012: Citeseer.

[12] Mizoguchi R, Kozaki K, Kou H, Yamagata Y, Imai T, Waki K, et al, editors. River Flow Model of Diseases ICBO; 2011.

[13] Rovetto RJ, Mizoguchi R. Causality and the ontology of disease. Applied Ontology. 2015;10(2):79–105.

[14] Yamagata Y, Kozaki K, Imai T, Ohe K, Mizoguchi R. An ontological modeling approach for abnormal states and its application in the medical domain. Journal of biomedical semantics. 2014;5(1):23. 24944781 10.1186/2041-1480-5-23 PMC4062306

[15] Campillos M, Kuhn M, Gavin A-C, Jensen LJ, Bork P. Drug target identification using side-effect similarity. Science. 2008;321(5886):263–6. 10.1126/science.1158140 18621671

[16] Chiang AP, Butte AJ. Systematic evaluation of drug–disease relationships to identify leads for novel drug uses. Clinical Pharmacology & Therapeutics. 2009;86(5):507–10. 19571805 10.1038/clpt.2009.103 PMC2836384

[17] Goh K-I, Cusick ME, Valle D, Childs B, Vidal M, Barabási A-L. The human disease network. Proceedings of the National Academy of Sciences. 2007;104(21):8685–90. 10.1073/pnas.0701361104 PMC1885563 17502601

[18] Hidalgo CA, Blumm N, Barabási A-L, Christakis NA. A dynamic network approach for the study of human phenotypes. PLoS computational biology. 2009;5(4):e1000353 10.1371/journal.pcbi.1000353 19360091 PMC2661364

[19] Lee D-S, Park J, Kay K, Christakis NA, Oltvai Z, Barabási A-L. The implications of human metabolic network topology for disease comorbidity. Proceedings of the National Academy of Sciences. 2008. 10.1073/pnas.0802208105 PMC2481357 18599447

[20] Zhang X, Zhang R, Jiang Y, Sun P, Tang G, Wang X, et al The expanded human disease network combining protein–protein interaction information. European Journal of Human Genetics. 2011;19(7):783 10.1038/ejhg.2011.30 21386875 PMC3137500

[21] Zhou X, Menche J, Barabási A-L, Sharma A. Human symptoms–disease network. Nature communications. 2014;5:4212 10.1038/ncomms5212 24967666

[22] Davis DA, Chawla NV. Exploring and exploiting disease interactions from multi-relational gene and phenotype networks. PloS one. 2011;6(7):e22670 10.1371/journal.pone.0022670 21829475 PMC3146471

[23] Yao X, Hao H, Li Y, Li S. Modularity-based credible prediction of disease genes and detection of disease subtypes on the phenotype-gene heterogeneous network. BMC systems biology. 2011;5(1):79. 21599985 10.1186/1752-0509-5-79 PMC3130676

[24] Wu X, Jiang R, Zhang MQ, Li S. Network‐based global inference of human disease genes. Molecular systems biology. 2008;4(1):189. 18463613 10.1038/msb.2008.27 PMC2424293

[25] Zhao S, Li S. Network-based relating pharmacological and genomic spaces for drug target identification. PloS one. 2010;5(7):e11764 10.1371/journal.pone.0011764 20668676 PMC2909904

[26] Park S, Lee D-g, Shin H. Network mirroring for drug repositioning. BMC medical informatics and decision making. 2017;17(1):55. 28539121 10.1186/s12911-017-0449-x PMC5444046

[27] Cormen TH, Leiserson CE, Rivest RL, Stein C. Introduction to algorithms: MIT press; 2009.

[28] Hart PE, Nilsson NJ, Raphael B. A formal basis for the heuristic determination of minimum cost paths. IEEE transactions on Systems Science and Cybernetics. 1968;4(2):100–7.

[29] Dijkstra EW. A note on two problems in connexion with graphs. Numerische mathematik. 1959;1(1):269–71.

[30] Floyd RW. Algorithm 97: shortest path. Communications of the ACM. 1962;5(6):345.

[31] Warshall S. A theorem on boolean matrices. Journal of the ACM (JACM). 1962;9(1):11–2.

[32] Yen JY. Finding the k shortest loopless paths in a network. management Science. 1971;17(11):712–6.

[33] Ahuja RK, Mehlhorn K, Orlin J, Tarjan RE. Faster algorithms for the shortest path problem. Journal of the ACM (JACM). 1990;37(2):213–23.

[34] Aljazzar H, Leue S. K⁎: A heuristic search algorithm for finding the k shortest paths. Artificial Intelligence. 2011;175(18):2129–54.

[35] Eppstein D. Finding the k shortest paths. SIAM Journal on computing. 1998;28(2):652–73.

[36] Wu Q, Hartley J. Using k-shortest paths algorithms to accommodate user preferences in the optimization of public transport travel. Applications of Advanced Technologies in Transportation Engineering (2004) 2004 p. 181–6.

[37] Galbrun E, Pelechrinis K, Terzi E. Urban navigation beyond shortest route: The case of safe paths. Information Systems. 2016;57:160–71.

[38] Carter H, Bhandari R, editors. Improved Sliding Shortest Path Algorithm: Performance Analysis. Proceedings of the Southeastern International Conference on Combinatorics, Graph Theory and Computing; 2011.

[39] Berclaz J, Fleuret F, Turetken E, Fua P. Multiple object tracking using k-shortest paths optimization. IEEE transactions on pattern analysis and machine intelligence. 2011;33(9):1806–19. 10.1109/TPAMI.2011.21 21282851

[40] Xi Z, Liu H, Liu H, Yang B. Multiple object tracking using the shortest path faster association algorithm. The Scientific World Journal. 2014;2014. 10.1155/2014/481719 PMC4151586 25215322

[41] Chen L, Hao Xing Z, Huang T, Shu Y, Huang G, Li H-P. Application of the shortest path algorithm for the discovery of breast cancer-related genes. Current Bioinformatics. 2016;11(1):51–8.

[42] Jiang M, Chen Y, Zhang Y, Chen L, Zhang N, Huang T, et al Identification of hepatocellular carcinoma related genes with k-th shortest paths in a protein–protein interaction network. Molecular BioSystems. 2013;9(11):2720–8. 10.1039/c3mb70089e 24056857

[43] Zhang J, Jiang M, Yuan F, Feng K-Y, Cai Y-D, Xu X, et al Identification of age-related macular degeneration related genes by applying shortest path algorithm in protein-protein interaction network. BioMed research international. 2013;2013. 10.1155/2013/523415 PMC3878555 24455700

[44] Chandrasekaran S, Bonchev D. A network view on Parkinson's disease. Computational and structural biotechnology journal. 2013;7(8):e201304004. 24688734 10.5936/csbj.201304004 PMC3962195

[45] Shih Y-K, Parthasarathy S. A single source k-shortest paths algorithm to infer regulatory pathways in a gene network. Bioinformatics. 2012;28(12):i49–i58. 10.1093/bioinformatics/bts212 22689778 PMC3371844

[46] Uhlmann V, Haubold C, Hamprecht FA, Unser M. DiversePathsJ: diverse shortest paths for bioimage analysis. Bioinformatics. 2017;34(3):538–40. 10.1093/bioinformatics/btx621 PMC5860364 29029024

[47] MeSH. Medical Subject Headings]. Available from: http://www.ncbi.nlm.nih.gov/mesh.

[48] PharmDB. Integrated database for diseases, proteins, and drugs]. Available from: http://www.pharmdb.org.

[49] CTD. Comparative Toxicogenomics Database]. Available from: http://www.ctdbase.org.

[50] GAD. Genetic Association Database]. Available from: http://www.geneticassociationdb.nih.gov.

[51] OMIM. Online Mendelian Inheritance in Man]. Available from: http://www.omim.org.

[52] PharmGKB. The Pharmacogenomics Knowledge Base]. Available from: http://www.pharmgkb.org. 10.1007/978-1-62703-435-7_20 PMC4084821 23824865

[53] KEGG. Kyoto encyclopedia of genes and genomes]. Available from: http://www.genome.jp/kegg/pathway.html.

[54] HuDiNe. Available from: http://hudine.neu.edu.

[55] PubMed. US National Library of Medicine National Institutes of Health]. Available from: http://www.nlm.nih.gov/databases/download/pubmed_medline.html.

