# Supplementary material for: Inference on chains of disease progression based on disease networks
Source: PLoS One. 2019 Jun 28;14(6):e0218871. doi: 10.1371/journal.pone.0218871 (PMC6599221; doi:10.1371/journal.pone.0218871)
Supplement: S1 Appendix — (PDF) [file pone.0218871.s002.pdf]

Appendix

Table A shows supplementary information of disease progression chains between colitis and respiratory insufficiency (an exemplary case in results for disease progression chain section) and additional progression chains. In the table, the notation $\{\mathrm{A} \rightarrow \mathrm{B}\}_{(i,j)}^{\{k\}}$ denotes the progression chain from disease A to B. $\{k\}$ indicates the kth chain, and (i,j) indicates a prior-posterior relation between the ith and jth diseases within the chain. For example, $\{\text{Colitis} \rightarrow \text{Respiratory Insufficiency}\}_{(1,2)}^{\{1\}}$ represents a progression from colitis to acute kidney injury in the first disease progression chain of colitis and respiratory insufficiency. The fifth column shows sources of progression with A, C, P, and T denoting association, clinical history, pathway, and text, respectively. The sixth column is the weight value of (i,j) in the integrated disease progression network.

Table A. Supplementary information of disease progression chains

$\{\mathbf{Colitis} \rightarrow \mathbf{Respiratory\ Insufficiency}\}_{(i,j)}^{\{k\}}$					
$\{k\}$	**CIS**	(i,j)	**Prior-Posterior Relation**	**Sources**	**Weight**
1	26.31	(1,2)	(Colitis → Acute Kidney Injury)	A, C	0.0510
		(2,3)	(Acute Kidney Injury → Polyuria)	A, C	0.1556
		(3,4)	(Polyuria → Hyponatremia)	A	0.0962
		(4,5)	(Hyponatremia → Respiratory Insufficiency)	A, C	0.0595
2	25.92	(1,2)	(Colitis → Diabetes Insipidus)	C	0.0118
		(2,3)	(Diabetes Insipidus → Polyuria)	A, C	0.1796
		(3,4)	(Polyuria → Hyponatremia)	A	0.0962
		(4,5)	(Hyponatremia → Respiratory Insufficiency)	A, C	0.0595
3	24.68	(1,2)	(Colitis → Polydipsia)	C	0.0139
		(2,3)	(Polydipsia → Polyuria)	C	0.1288
		(3,4)	(Polyuria → Hyponatremia)	A	0.0962
		(4,5)	(Hyponatremia → Respiratory Insufficiency)	A, C	0.0595
4	4.78	(1,2)	(Colitis → Acute Kidney Injury)	A, C	0.0510
		(2,3)	(Acute Kidney Injury → Oliguria)	A, C	0.1774
		(3,4)	(Oliguria → Diabetes Insipidus)	A, C	0.0939
		(4,5)	(Diabetes Insipidus → Polyuria)	A, C	0.1796
		(5,6)	(Polyuria → Hyponatremia)	A	0.0962
		(6,7)	(Hyponatremia → Respiratory Insufficiency)	A, C	0.0595
5	4.39	(1,2)	(Colitis → Renal Colic)	C	0.0038
		(2,3)	(Renal Colic → Oliguria)	A, C	0.1379
		(3,4)	(Oliguria → Diabetes Insipidus)	A, C	0.0939
		(4,5)	(Diabetes Insipidus → Polyuria)	A, C	0.1796
		(5,6)	(Polyuria → Hyponatremia)	A	0.0962
		(6,7)	(Hyponatremia → Respiratory Insufficiency)	A, C	0.0595
$\{\mathbf{Arthritis, Rheumatoid} \rightarrow \mathbf{Dementia}\}_{(i,j)}^{\{k\}}$					
$\{k\}$	**CIS**	(i,j)	**Prior-Posterior Relation**	**Sources**	**Weight**
1	18.48	(1,2)	(Arthritis, Rheumatoid → Hypertension)	A, P	0.3095
		(2,3)	(Hypertension → Dementia)	C	0.0018
2	17.82	(1,2)	(Arthritis, Rheumatoid → Diabetes Mellitus, Type 2)	A, P	0.2714
		(2,3)	(Diabetes Mellitus, Type 2 → Dementia)	C	0.0040
8	8.72	(1,2)	(Arthritis, Rheumatoid → Graft vs Host Disease)	A, P	0.3078
		(2,3)	(Graft vs Host Disease → Diabetes Mellitus, Type 2)	P	0.2483
		(3,4)	(Diabetes Mellitus, Type 2 → Dementia)	C	0.0040
10	8.60	(1,2)	(Arthritis, Rheumatoid → Diabetes Mellitus, Type 2)	A, P	0.2714
		(2,3)	(Diabetes Mellitus, Type 2 → Hypertension)	A, P	0.2736
		(3,4)	(Hypertension → Dementia)	C	0.0018
$\{\mathbf{Hyperlipidemias} \rightarrow \mathbf{Myocardial\ Infarction}\}_{(i,j)}^{\{k\}}$					
$\{k\}$	**CIS**	(i,j)	**Prior-Posterior Relation**	**Sources**	**Weight**
1	16.30	(1,2)	(Hyperlipidemias → Thrombosis)	C	2.51×10^{-5}
		(2,3)	(Thrombosis → Myocardial Infarction)	A, T	0.1856
2	13.56	(1,2)	(Hyperlipidemias → Coronary Occlusion)	C	9.07×10^{-5}
		(2,3)	(Coronary Occlusion → Myocardial Infarction)	T	0.0016
6	7.35	(1,2)	(Hyperlipidemias → Glycogen Storage Disease)	C	3.41×10^{-5}
		(2,3)	(Glycogen Storage Disease → Hypertension)	A, P	0.3021
		(3,4)	(Hypertension → Myocardial Infarction)	A, C	0.0879
11	6.46	(1,2)	(Hyperlipidemias → Neuritis)	C	3.25×10^{-5}
		(2,3)	(Neuritis → Herpes Zoster)	A, C	0.2599
		(3,4)	(Herpes Zoster → Myocardial Infarction)	T	1.61×10^{-4}
